# Partnership Choice and Childbearing in Norway and Spain

**DOI:** 10.1007/s10680-017-9432-6

**Published:** 2017-06-22

**Authors:** Roberta Rutigliano, Gøsta Esping-Andersen

**Affiliations:** Department of Political and Social Sciences, Universitat Pompeu Fabra, Ramon Trias y Fargas Street, 25-27, 08005 Barcelona, Spain

**Keywords:** Fertility, Cohabitation, Marriage, Simultaneous equation models, Spain, Norway

## Abstract

Cohabitation has, in a number of countries, become a genuine alternative to marriage. Where this occurs, will we see a convergence in fertility behavior between the two partnership options? We address this question by comparing two societies, Norway and Spain, that contrast sharply not only in the evolution of cohabitation, but also in overall birth rates and public support for families. Using the Generations and Gender Survey for Norway (2007/2008) and the most recent Fertility, Family and Values Survey for Spain (2006), we estimate a three-equation multi-process model for selection into a union and fertility in order to take into account unobserved heterogeneity. For Norway, we find a significant association between selection into either partnership type and fertility, whereas for Spain, a newcomer to cohabitation, we find a significant association between fertility and selection into marriage.

**Electronic supplementary material:**

The online version of this article (doi:10.1007/s10680-017-9432-6) contains supplementary material, which is available to authorized users.

## Introduction

Demographic research has produced no clear evidence as regards the influence of partnership types on fertility. The Second Demographic Transition thesis sees cohabitation as a key marker of postmodern values which stress individualism and self-realization (Lesthaeghe 2010). In this framework, one would assume that cohabitation is a favored option among those who are less inclined to enter into long-term and binding commitments.

Historically, there is a close fit between the surge in divorce and cohabitation—although here Latin America is an exception (Laplante et al. 2015). Cohabitation gained ground especially in high-divorce societies, like Scandinavia and France, while remaining more marginal in low-divorce settings, like Italy.^1^ If cohabitation represents weaker commitments, one would expect it to be associated with lower fertility. But is that necessarily the case?

There are three reasons why we should question this prediction. Firstly, the link between couple (in) stability and fertility is inherently ambiguous. We would expect that stable partnerships are more likely to have children. And yet, couples may also have children as a way to shore up a shaky relationship. There is empirical support for both views (Malpas and Lambert 1993; European Commission 1997; Testa 2007). Similar findings emerge for Germany (Berninger et al. 2011). Earlier US studies found that the risk of relationship disruption decreases the likelihood of births (Lillard and Waite 1993; Myers 1997; Manning 2004), and this appears also to hold for Italy and Spain (Coppola and Cesare 2008). Union stability also predicts higher overall fertility in France (Thomson et al. 2012) and in the Netherlands (Rijken and Thomson 2011).

The ‘births induce stability’ perspective argues that childbearing, given that it is irreversible and shared, increases marital satisfaction and strengthens relationships (Lillard and Waite 1993). This argument, too, enjoys empirical support. Relationships stabilize after the first or early higher-parity births in the USA (Waite and Lillard 1991) as well as in Italy and Spain (Coppola and Cesare 2008). Steele et al. (2007) compare across two UK cohorts (born 1958 and 1970, respectively) and find that births solidify cohabiting relationships within the younger, but not the older, cohort (see also Rijken and Liefbroer 2009).

The second reason lies in the multifaceted nature of cohabitation. In some societies, like Germany, the USA or UK, it is largely a temporary testing ground prior to committing oneself, or simply an alternative to singlehood (Rindfuss and VandenHeuvel 1990; Hiekel et al. 2014); in others, like France and Scandinavia, it has become a de facto equivalent to marriage (Raley 2001; Kiernan 2002). To this, we should add that Scandinavian cohabitation includes also a lot of ‘shacking up’ among young adults and, furthermore, cohabiters often marry after the birth of the first child. Youth emancipation from the parental home occurs exceptionally early here.^2^

All told, we would assume that fertility in cohabitation and marriage will begin to converge the more that cohabitation becomes normative and legally sanctioned. This is how Kiernan (2001) defines its mature state. And yet, the link between the diffusion of cohabitation and fertility may not be linear. As Perelli-Harris (2014) concludes, it is more likely curvilinear: as cohabitation becomes normatively enshrined, it is associated with lower fertility compared to married couples. This is explained by a selection effect: those (ever fewer) who opt for marriage from the start are more likely to espouse more traditional family values.

This raises an important point, namely that fertility differentials between cohabiting and married couples are likely to be driven by underlying selection mechanisms. There are surprisingly few studies which address this conundrum explicitly (an exception is Steele et al. 2005).

The third reason is that citizens may select themselves into cohabitation for reasons other than reluctance to commit themselves. Motives may be pecuniary, such as avoiding double taxation; an anticlerical ideology in societies where marriage is closely associated with the church (Dominguez-Folgueras and Castro-Martín 2013); or the embrace of postmodernist values so much stressed by Lesthaeghe (2010); finally, any given person’s choice may simply be a function of what significant others in his–her social environment do.

## A Norwegian–Spanish Comparison

We analyze fertility within cohabiting and married couples, comparing Norway and Spain which represent the polar ends as regards European fertility, with Norway at the high end (a quite stable TFR around 1.9–2.0) and Spain with lowest-low fertility (for more than two decades, the Spanish TFR has been below 1.4).

Our choice of comparison was, however, primarily motivated by clear contrasts in the two countries’ cohabitation and divorce profiles. Norway represents the Scandinavian model where cohabitation has been firmly entrenched for many decades. And Norway exhibits relatively high and stable divorce rates (a crude divorce rate of ca. 2.3).

**Fig. 1:**
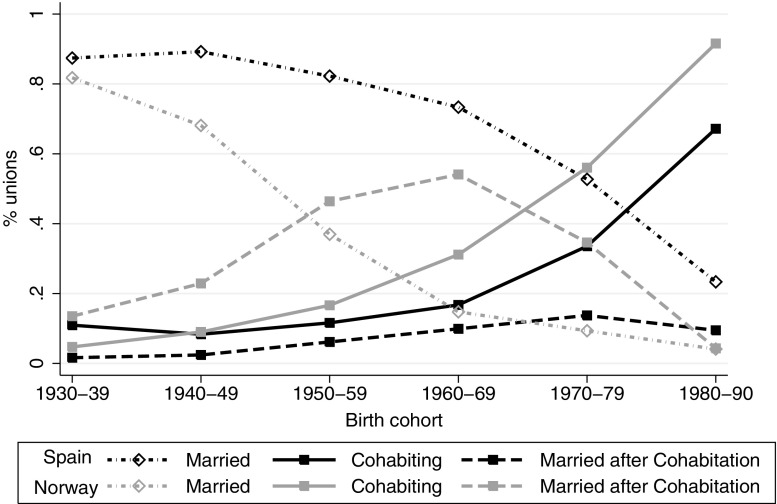
Type of union trends (as percent of all unions). Estimated from GGS (FFVS for Spain) using weights

Spain is a newcomer on both counts. And, yet, the pace of change has been truly explosive. Since divorce was legalized in 1981, Spain has moved from the bottom to the top in the divorce league (from a CDR of 0.5 in 1990 to 2.2 in 2010).^3^ In tandem, cohabitation rose from practically nil in 1990 to 17% of all unions in the mid-2000s. This might lead us to think that Spain represents the phase in which cohabitation is a response to rising union instability (Fig. 1).

**Fig. 2:**
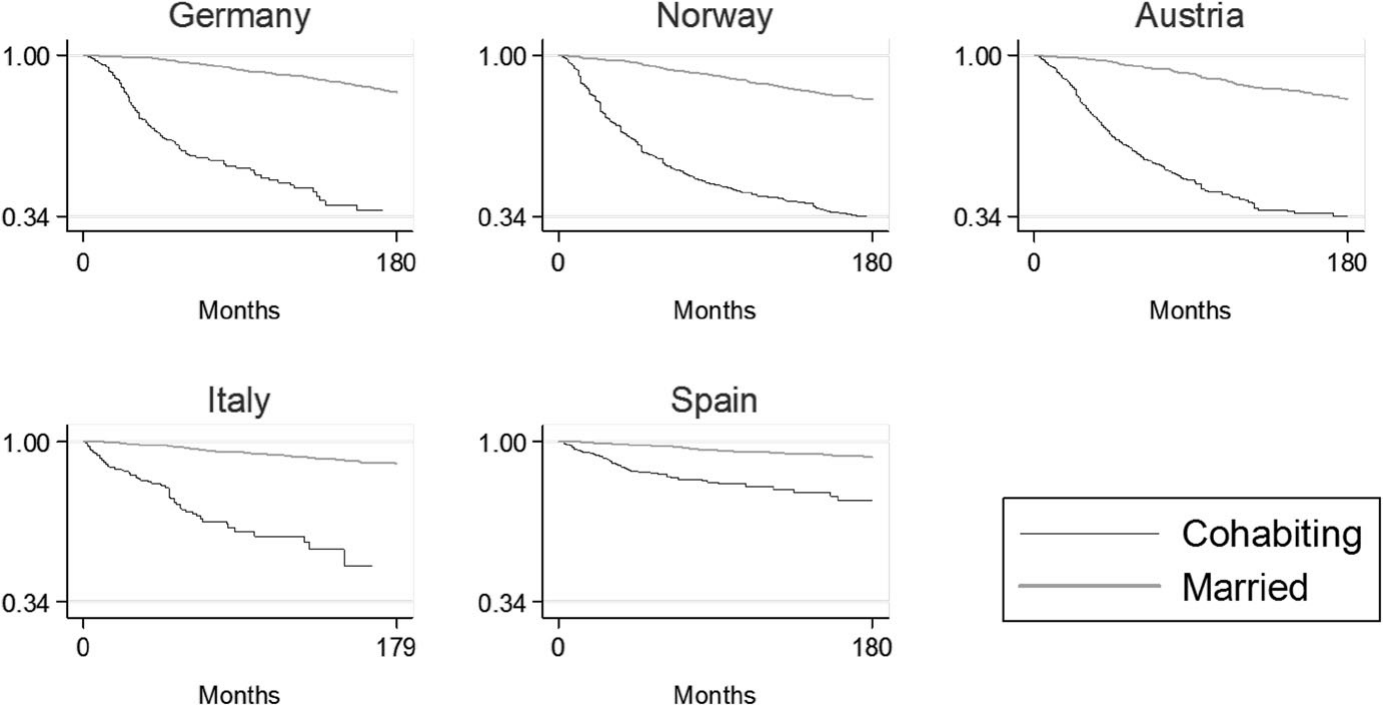
Kaplan–Meier survival curve for cohabiting and married couples (event = divorce/separation). *Source*: GGS data and the 2006 FFVS survey for Spain

However, Spanish cohabiters are extraordinarily stable: after 180 months of partnering, the share of intact couples is about twice as large as in Norway (or elsewhere; See Fig. 2). This suggests that the Spanish cohabitation boom may not be fueled by any reluctance to commit.^4^ In this regard, Spain, at first glance, appears more Scandinavian than even the Scandinavians can muster. Nonetheless, Spain follows a very different path to cohabitation. As Vitali et al. (2015) show, the rise of women’s education has been the principal driver in the diffusion of childbearing within cohabitation in Norway. In contrast, Spanish fertility within cohabitation is not related to education for the cohort born after 1960 (Dominguez-Folgueras and Castro-Martín 2013).

In Norway, cohabitation is well enshrined, both normatively and legally. It comprises, however, two very different logics: on the one hand, a large proportion of (mainly young) partnerships that tend to be short lived and, on the other hand, more mature and long-lasting arrangements in which childbearing is common (Wiik et al. 2009; Lyngstad et al. 2010). In Spain, cohabitation has become socially accepted, but it still does not enjoy the degree of legal sanctioning that marriage does (Dominguez-Folgueras and Castro-Martín 2013).

Spain represents therefore an interesting case. In terms of cohabitation, it clearly deviates from the Catholic-dominated Southern European ‘familialist’ model, as depicted in Reher (1998) and, more recently, in Perelli-Harris (2014). The features of Spanish cohabitation are partially related to late independence and to the difficulties of gaining a foothold in the labor market. Leaving the parental home usually coincides with union and family formation.^5^ But unique to the Spanish case is the intense secularization experienced after the Franco dictatorship (Requena 2005), which undoubtedly drives some segments of the population to favor cohabitation (see also Dominguez-Folgueras and Castro-Martín 2013). The differences and similarities in the two countries raise two questions. One, what are the mechanisms that select citizens into cohabitation? And, two, how do they influence childbearing?

One would, all else constant, expect that birth propensities within married and cohabiting unions will converge as the latter become broadly diffused across the population. But where cohabitation is viewed as little more than a trial partnership, we should expect a substantial fertility gap between the two (Kiernan 2001; Perelli-Harris 2014; Raley 2001; Heuveline and Timberlake 2004).

Considerable evidence supports this. Non-marital childbearing has risen in almost all advanced nations (Billari and Kohler 2004; Buchmann and Kriesi 2011). Nonetheless, cohabiting couples may be less likely to become parents (Brien et al. 1999; Spéder and Kapitány 2009). Here nation differences are substantial: in the USA and Germany, cohabiting couples have a significantly lower probability of giving birth; in France and Scandinavia there is no real difference (Baizan et al. 2003; Heaton et al. 1999; Heuveline and Timberlake 2004; Toulemon and Testa 2005).

**Fig. 3:**
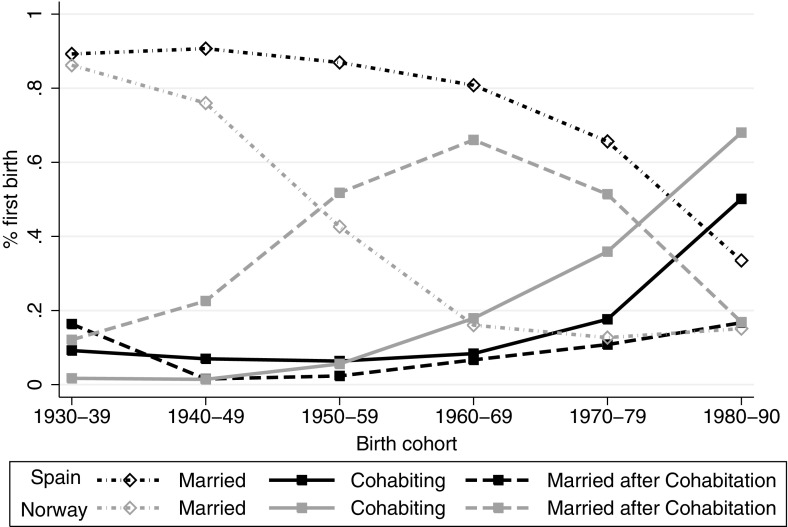
First birth by type of union (as percent of all first births). Estimated from GGS (FFVS for Spain) using weights

**Fig. 4:**
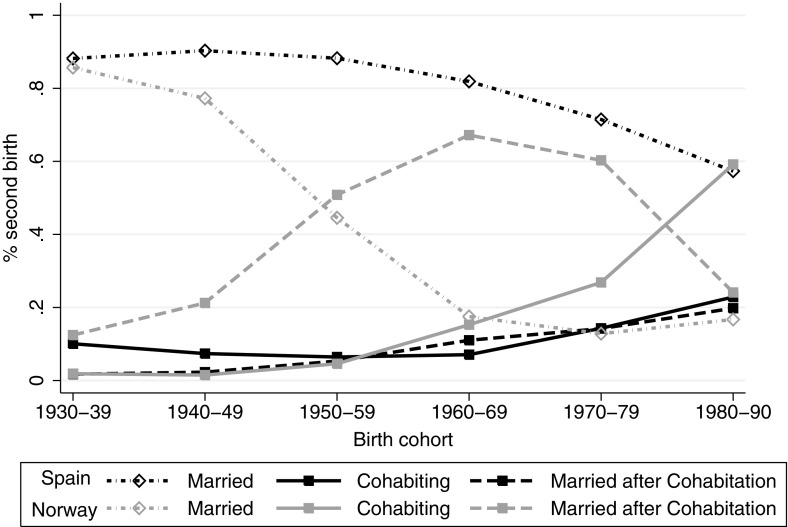
Second birth by type of union (as percent of all second births). Estimated from GGS (FFVS for Spain) using weights

Figures 3 and 4 show, for Norway and Spain, the proportion of first and second births in each type of union. The graphs are based on the weighted sample for the sake of representativeness. We distinguish three cases: permanent cohabitation, marriage and cohabitation followed by marriage.

Figure 3 shows the proportion of first births in each type of union across six birth cohorts. In Norway, apart from the two oldest cohorts, between 40 and 70% of first births occur within either premarital cohabitation or cohabitation. For the younger cohorts, marriage accounts for a minority of births (about 20%). This is pretty much in line with previous studies which show that Norwegian cohabiting couples with one child account for 40–50% of all in 2000. However, here cohabiting couples are four times more likely to break up than are married couples (Hyggen and Skevik 2002).

In Spain, except for the youngest cohort, marriage remains the main channel for first births. And yet, over the past decades the share of births in marriage has dropped from 90 to almost 65%. In parallel, first births within cohabitation have risen from nil to 30%. And within the youngest cohort, we observe that first births in cohabitation now exceed those in marriage.^6^

Figure 4 shows the proportion of second births for each type of union across six birth cohorts. In Spain, marriage is the principal option for second births. This has decreased over time but still accounts for about 60% of all births. As regards cohabiting couples, we see a slight increase in second births over the recent decades.

Note that the data in Fig. 4 do not take into account successive unions. It is possible that a second child was conceived with the same or with a different partner. Norway exhibits a completely different pattern. Here it is far more likely that the second birth will also occur within cohabitation. Indeed, for the youngest cohort, only 20% of second births occurred in marriage.

## Methods

Union formation and childbearing can be mutually related. Individuals may partner because they desire to have children or, reversely, they decide to form a union because they already expect a child. Furthermore, unobservable characteristics such as preferences or peer group influence may drive both processes. Applying multi-process models to event history data is a powerful tool in such situations (Steele et al. 2005). The advantage lies in their ability to provide unbiased estimates of the covariates by taking into account both selection on time-invariant unobservables and correlations across different processes (via random effects correlation—see below).

Unbiased estimates, however, do not come without a price since we are compelled to make a number of assumptions. Firstly, in each equation we assume that the vector of covariates *X* (only those that are not related to either fertility or partnership) is exogenous, i.e., not correlated with either the level one residual or with the level 2 random effect.

Our model includes two main components: the first addresses partnership formation and the second fertility transitions. We estimate partnering with two different competing risk models. The first estimates the risk of entering into either cohabitation or marriage for a single woman. The second estimates the risk of marriage with (or separation from) the same partner for cohabiting women. In other words, we take into account both those who change status from *single* to *married/cohabiting* and those who, after entry into cohabitation, marry the same partner or exit from cohabitation.

Selection into either outcome may be driven by individual characteristics (e.g., preferences). For instance, those who marry may see marriage as more stable than those who remain cohabiting. The variance–covariance matrix gives us a measure of the correlation between these processes which, in turn, helps us to better interpret these relationships. In modeling partnership dynamics, we take into account repeated events, i.e., those partnerships a woman experiences in life as well as possible partnership ruptures. A woman who experiences a divorce will automatically be assigned the status of singlehood.

Similarly to Steele et al. (2004), we define the competing risk of partnering for a single woman as follows. We denote by r the type of union in which individual *j* enters at each *t*th month (of episode *i*), where *r*_1_ = 1 is cohabitation and *r*_1_ = 2 is marriage. To estimate the first equation, we use a competing risks framework, with *h*_*ij*_^( *r*1)^ representing the hazard for a woman j, in the state of singlehood, of experiencing the transition of type r_1_, at the time spell *i* of the month *t*.^7^ The risk of a transition from single to each *r*_1_ state (from single to marriage, cohabitation, or remaining single), given the condition that no event has occurred before, can be written as:

1 $$\log \left( {\frac{{h_{ij}^{{\left( {r1} \right)}} }}{{h_{ij}^{\left( 0 \right)} }}} \right) = \alpha^{{\left( {{\text{r}}1} \right)}} {\mathbf{D}}_{ij}^{{\left( {r1} \right)}} + \beta^{{\left( {{\text{r}}1} \right)}} x_{ij}^{{\left( {r1} \right)}} + u_{j}^{{P \left( {r1} \right)}}$$

$$u_{j}^{{P \left( {r1} \right)}} \sim N\left( {0,\varvec{\varOmega}^{R1} } \right)\quad {\text{where}}\,R_{1} = 2$$

where *α* ^(*r*1)^ *D*_*ij*_^( *r*1)^ is a function of the duration of the state as ‘single’ and $x_{ij}^{{\left( {r1} \right)}}$ is a vector of covariates. $u_{j}^{{P \left( {r1} \right)}}$ is the random effect at the individual level. The dependent variable assumes the value of 1 when women either marry or start cohabitation and 0 if they remain single.

In the second model, transitions are from the state of ‘cohabiting’ to each r state ‘married’ (with the same partner) or ‘separated.’ Thus, adopting the same notation of Eq. (1), the hazard *h*_*ij*_^( *r*2)^ for a woman *j*, of experiencing the transition of type *r*_2_, at the time spell *i* of the month *t* may be expressed as:

2 $$\log \left( {\frac{{h_{ij}^{{\left( {r2} \right)}} }}{{h_{ij}^{\left( 0 \right)} }}} \right) = \alpha^{{\left( {{\text{r}}2} \right)}} {\mathbf{T}}_{ij}^{{\left( {r2} \right)}} + \beta^{{\left( {{\text{r}}2} \right)}} c_{ij}^{{\left( {r2} \right)}} + v_{j}^{{P \left( {r2} \right)}}$$

$$v_{j}^{{P \left( {r2} \right)}} \sim N\left( {0,\varvec{\varOmega}^{R2} } \right)\quad {\text{where}}\,R_{2} = 2$$

where *α* ^(*r*2)^ *T*_*ij*_^( *r*2)^ is a function of the duration of the state as a ‘cohabiting’ and $c_{ij}^{{\left( {r2} \right)}}$ is a vector of covariates. $v_{j}^{{P\left( {r2} \right)}}$ is the individual-level random effect. The dependent variable is dichotomous, equal to one when women either marry their cohabiting partner or separate, or zero if they remain cohabiting.

The second main component of the model focuses on fertility transitions from the date of union formation onwards.^8^ Our aim is to identify whether different union types exhibit different normative perceptions via both the coefficients and the variance–covariance matrix. We limit our analyses to first and second births because we expect that the transition to the second is crucial. We include both births in a unique equation and treat them as repeated events. Parities are defined in terms of a woman’s fertility history, regardless of re-partnering.

This entails that a woman can have her first and second birth with two different partners. In fact, in higher-order relationships, women may self-select into a different type of union compared to women who enter into their first. It has been shown that re-partnered mothers are more likely to opt for cohabitation (Heuveline and Timberlake 2004). In contrast, women who have both the first and second child with the same partner may represent a different kind of self-selection.

Further, since the same covariate may have a different effect at different parities, we include one indicator variable for each parity, interacting it with every other covariate in the model as well as with the duration function. This implies that we have two parallel embedded equations.

Formally, by denoting *h*_*ij*_^*F*^ as the risk of a birth for the woman *j* in her *i*th episode, in the *t*th month, the two-level random effects logit model can be written as:

3 $$\log \left( {h_{ij}^{F} } \right) = \log \left( {\frac{{p_{ijt}^{F} }}{{1 - p_{ij}^{F} }}} \right) = \left[ {\alpha^{F} G_{ij}^{F} + \gamma^{F} \,_{0} k_{ij}^{F} + \beta^{F} \,_{1} m_{ij}^{F} } \right]*(1 - c_{ij}^{F} ) + \left[ {\alpha^{F} G_{ij}^{F} + \gamma^{F} \,_{1} k_{ij}^{F} + \beta^{F} \,_{2} m_{ij}^{F} } \right]*c_{ij}^{F} + e_{j}^{F}$$

$$e_{j}^{F} \sim N\left( {0,\sigma_{C}^{2} } \right)$$

Here $m_{ij}^{F}$ is a vector of covariates including birth cohort, education level and background characteristics of the women (i.e., country of birth, whether they experienced partnership dissolutions, whether their parents separated before the age of 16, and age at partnership). $k_{ij}^{F}$ is a dummy identifying the current type of union at each spell *i*; it is one for married couples and zero for cohabiters. $c_{ij}^{F}$ is a dummy for parities that equal zero for childless women and one for those who already have a child. Finally, *c*_*ij*_^*F*^ * *p*_*ij*_^*F*^ and *c*_*ij*_^*F*^ * *k*_*ij*_^*F*^ are the interactions identifying, respectively, the effect of the covariates for each birth order and type of union in which each birth transition occurs. This allows us to isolate the effect of the covariates for every fertility transition within each type of union, as the same covariates may influence the first and second birth differently. The dependent variable is binary, assuming the value of one when the partnered woman experiences a first, respectively, second birth.

**Fig. 5:**
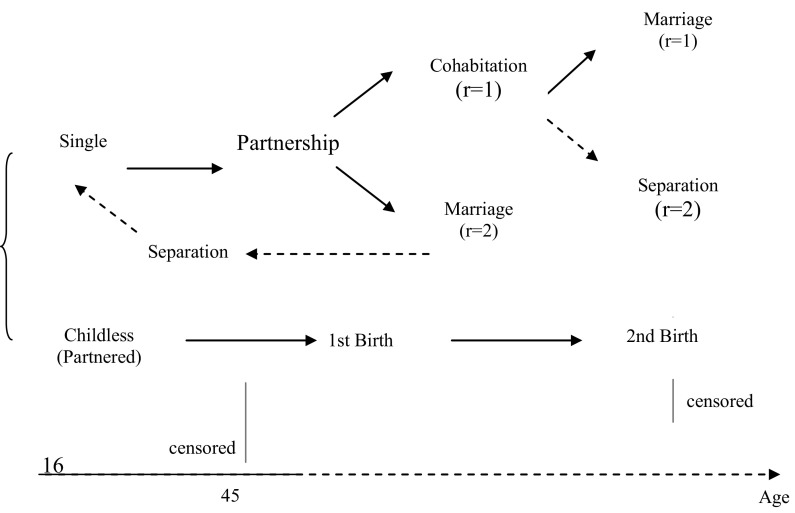
Full model

To sum up, in Eq. 1 women may partner, be it in cohabitation or marriage, in every spell of singlehood. The second equation estimates fertility transitions within partnerships.^9^ Figure 5 depicts the full multi-process model.

We include an individual random effect that allows us to identify selection, i.e., unobserved heterogeneity shared between the different processes. The model allows these individual-level random effects to be correlated across equations. From these, we obtain a variance–covariance matrix which informs us about the interrelation between the different processes and also about the level of unobserved heterogeneity within each. The diagonal represents the variance which, if statistically significant, can be interpreted as the presence of unobserved heterogeneity at the woman level. In the lower quadrant, we find covariance estimates which represent the correlation between different processes due to unobserved heterogeneity. This matrix provides us with a measure that would be unavailable using other methods. It allows us to interpret the coefficients of the regression model by providing both a sign and a direction of the correlation between selection into different processes. Note, however, that we must assume that attitudes and preferences, not captured by control variables, are time invariant and also normally distributed.^10^

## Data

For Norway, we use the Generations and Gender Survey (GGS) 2007/2008; for Spain, the Fertility, Family and Values Survey (FFVS) from 2006—the best recent source of data for Spain. Both include retrospective information that allows intergenerational and longitudinal analysis. We include all women in their reproductive years (15–45), censoring at their 45th birthday or at the second birth.^11^ We follow women born 1960–1990. In Spain, these cohorts coincide with the surge in cohabitation. Since we also focus on changes in the type of union between births, we select all partnered women for whom we may observe a first and second birth. For the fertility equation, our sample size is 2797 for Spain and 3142 for Norway.

Appendix Tables A1–A4 of the Electronic Supplementary Material present descriptive statistics for the fertility equation sample. Although we focus only on recent cohorts, we present descriptive statistics for older birth cohorts (born 1930–1959) to trace how selection into different types of unions changes over time. In order to properly identify shifts in partnering behavior across the different cohorts, we should have estimated two models for the two periods. However, due to the low number of cohabitants in the old cohorts, multi-process models cannot be identified. We include the standard covariates in the partnering models; the fertility equation, which is our main focus, includes the following covariates:

- parental divorce before the age of 16; country of birth, age at partnership and possible previous partnerships (time varying).
- A set of dummies for level of education (including a category for missing values in order not to lose too many observations). In both countries, the intermediate category (‘upper secondary’) is the largest. Dummies for birth cohort and current partnership duration (time varying) are included. As to the former, the distribution by birth cohort is fairly homogeneous in both countries.

We model each process with a discrete-time hazard. We estimate a two-level, random intercept logit, the two levels corresponding to the random effects related to different time spells for the same individual and to the random effects between women. Durations are grouped into 6-month intervals if no event occurs. The results are obtained using MCMC estimation in MLwiN through STATA 13 with the *runmlwin* command (Leckie and Charlton 2013).

## Results

For the sake of brevity, we shall focus on the variance–covariance matrix and on predicted probabilities, and when needed, we highlight the difference in estimates between multi- and single-process estimates.^12^ Results for the partnership equations are presented in the online Appendix (Tables A5 and A6).

The key difference between the single- and multi-process models is that the latter reduces the risk of estimation bias. The relationships are summarized in the variance–covariance matrix for Spain and Norway, respectively (Tables 1 and 2). The variance represents individual-level heterogeneity, and the covariance (on the sub-diagonal) identifies selection dynamics.

**Table 1:** Random effects variance–covariance matrix from the multi-process models for Spain

	Single to married	Single to cohabitating	Cohabiting to married	Cohabiting to separated	Fertility transition
Single to married	0.82***				
	[0.22]				
Single to cohabiting	0.08	1.97***			
	[0.16]	[0.29]			
Cohabiting to married	0.10	−0.40*	1.24***		
	[0.17]	[0.20]	[0.33]		
Cohabiting to separated	0.56**	−0.59+	0.71+	3.08**	
	[0.27]	[0.31]	[0.39]	[1.02]	
Fertility transition	0.17*	−0.07	0.64***	0.58**	0.81***
	[0.07]	[0.08]	[0.11]	[0.21]	[0.05]

Standard errors in brackets ^+^ *p* < 0.1; * *p* < 0.05; ** *p* < 0.01; *** *p* < 0.001

**Table 2:** Random effects variance–covariance matrix from the multi-process model for Norway

	Single to married	Single to cohabitating	Cohabiting to married	Cohabiting to separated	Fertility transition
Single to married	1.93***				
	[0.47]				
Single to cohabiting	−0.07	0.46***			
	[0.13]	[0.06]			
Cohabiting to married	0.04	0.08^+^	0.60***		
	[0.14]	[0.04]	[0.10]		
Cohabiting to separated	0.06	0.08+	0.35***	0.28**	
	[0.18]	[0.04]	[0.06]	[0.09]	
Fertility transition	−0.02	0.13***	0.69***	0.44***	0.91***
	[0.10]	[0.04]	[0.06]	[0.07]	[0.05]

Standard errors in brackets ^+^ *p* < 0.1; * *p* < 0.05; ** *p* < 0.01; *** *p* < 0.001

For Spain, we observe a substantial degree of unobserved heterogeneity driving all five processes. We find a negative association (−0.40) between entry into cohabitation and subsequently getting married. Similarly, those women who have an above-average risk of getting married have also an above-average risk of exiting cohabitation. This may reflect different unobservable characteristics—for instance, those who get married may experience shorter cohabitation periods. Turning to fertility, we find a positive and slightly significant correlation between entry into marriage and fertility (0.17). Additionally, we find a positive and significant coefficient (0.64) for the interrelation between fertility transitions and entry into marriage (with the same partner) after cohabitation.

This suggests that individuals with an above-average risk of getting married have also an above-average risk of childbirth. We find a negative (but not significant) correlation between fertility and entry into cohabitation from the status of single (−0.07). In other words, women who select themselves into cohabitation do not exhibit a stronger childbirth propensity (unless the cohabitation leads to marriage).

To summarize: first, entry into parenthood is endogenous with respect to entry into marriage; second, women who are more likely to enter into marriage share unobserved characteristics with those who are more likely to have children. It is important to note that we find no significant negative correlation between entry into cohabitation and childbearing. Women who select themselves into cohabitation are more likely to have children only if that cohabitation precedes a marriage. This is a crucial finding for understanding the Spanish context in terms of childbirth within cohabitation.

For Norway, the main diagonal shows statistical significance throughout, implying the presence of individual heterogeneity behind all processes. This underscores the relevance of our modeling approach.

The fertility coefficients suggest that women who want children are also more likely to enter into any type of union except marriage. In particular, we find a positive relation between fertility and conception within both cohabitation (0.13) and premarital cohabitation (0.69). Here we observe a major nation–contrast, because Norway exhibits a positive and significant correlation between fertility and entry into cohabitation. This difference may reflect the different degrees of acceptance of childbirth within cohabitation. In other words, Norwegian women do not perceive a non-marital birth as normatively deviant.

Moving now to the estimations, our primary interest lies in the coefficient for union type at each birth transition. Tables 3 and 4 report estimated coefficients for type of union at first and second birth for Spain and Norway. In order to highlight the appropriateness of our method, we present coefficients from the multi-process estimation along with the single-process results.

**Table 3:** MCMC estimation for childbirth within partnership (single versus multi-process) for Spain

	Single process	Multi-process
Childless women		
Constant	−4.20***	−3.72***
Marriage (ref. Cohab)	1.07***	0.62***
One-child women		
Constant	−6.12***	−5.66***
Marriage (ref. Cohab)	−0.51***	−0.50***

Significance levels ^+^ *p* < 0.1; * *p* < 0.05; ** *p* < 0.01; *** *p* < 0.001

**Table 4:** MCMC estimation for childbirth within partnership (single versus multi-process) for Norway

	Single process	Multi-process
Childless women		
Constant	−3.94***	−3.57***
Marriage (ref. Cohab)	0.70***	0.66***
One-child women		
Constant	−5.05***	−5.09***
Marriage (ref. Cohab)	−0.17*	−0.53***

Significance levels ^+^ *p* < 0.1; * *p* < 0.05; ** *p* < 0.01; *** *p* < 0.001

In both single- and multi-process estimations for Spanish married childless women, the coefficient in the multi-process is smaller. This is because we have ‘cleansed’ the coefficient of the positive correlation between fertility transitions and marriage; without allowing for this correlation, we would have overstated the effect of marriage on childbirth.

Turning to second births, the coefficient for married women is negative in both single- and multi-process estimation. Further, the difference in terms of size of the two coefficients is almost zero. This can be explained by the fact that second births in the more recent Spanish cohorts are less common.^13^

**Fig. 6:**
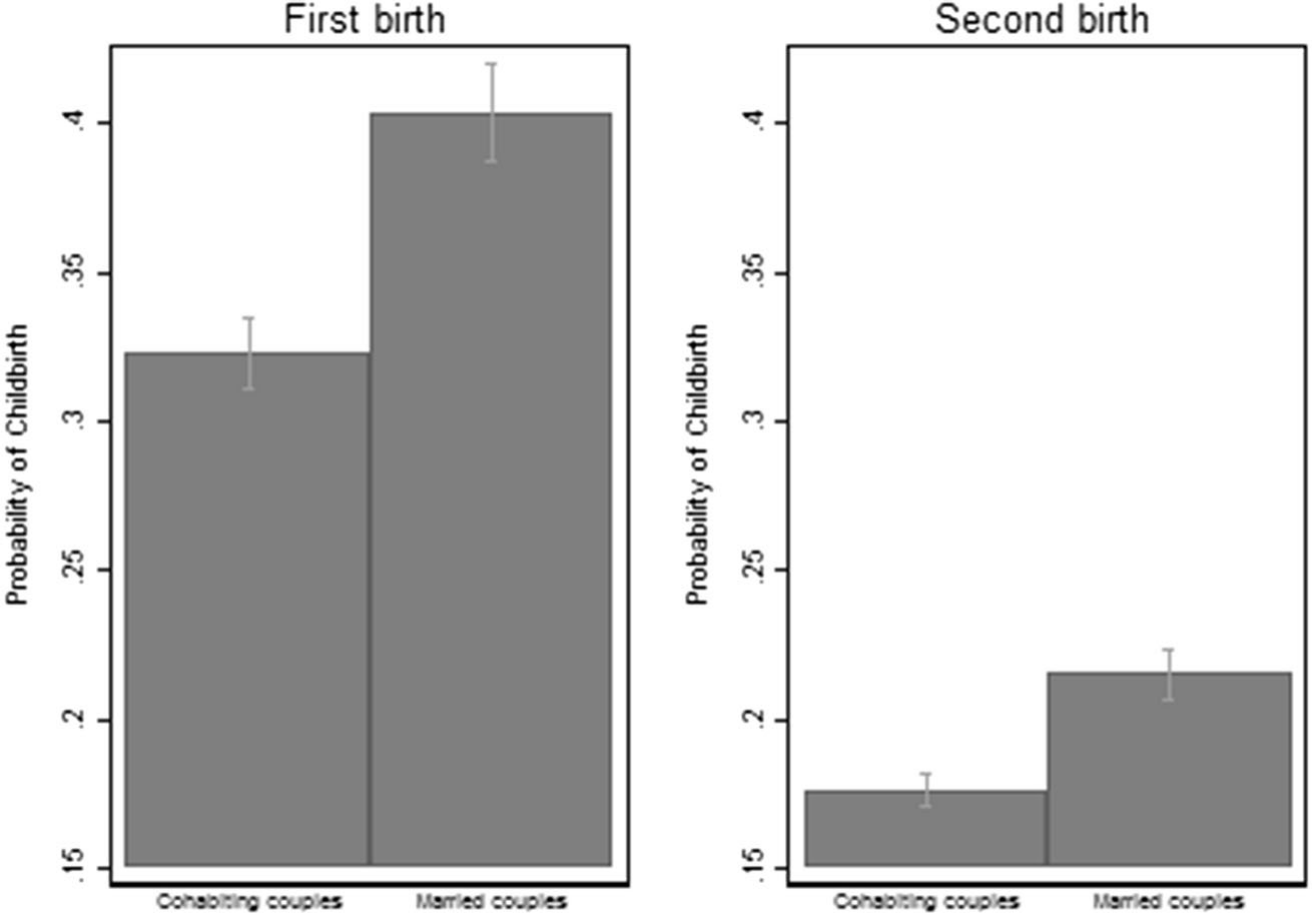
Spain-predicted probability of first and second birth by type of union (multi-process estimates)

To sum up, when we examine predicted probabilities for both first- and second-birth transitions in Spain (Fig. 6), married women are more likely than cohabiting women to give birth. However, when it comes to second births, the predicted probabilities are way smaller. Here we must remember that second births in Spain, especially for this cohort, are less common.

Turning to Norway, we see that the coefficient for first births is quite similar in both models (slightly lower in the multi-process estimation). One possible explanation is that first births are common in both types of union, so controlling for time-invariant unobservables makes little difference.^14^

Married women at risk of a second birth show a negative coefficient in both the single- and multi-process model. In the latter, the coefficient is smaller than in the single-process estimation. This is partially in line with what we found in the variance covariance matrix. On the one hand, women who are keener to have a child are less likely to get married. On the other hand, those who are more likely to marry their cohabiting partner are also more likely to experience childbirth. Accordingly, in the single-process approach the marriage effect is exaggerated because of selection on unobservables.

**Fig. 7:**
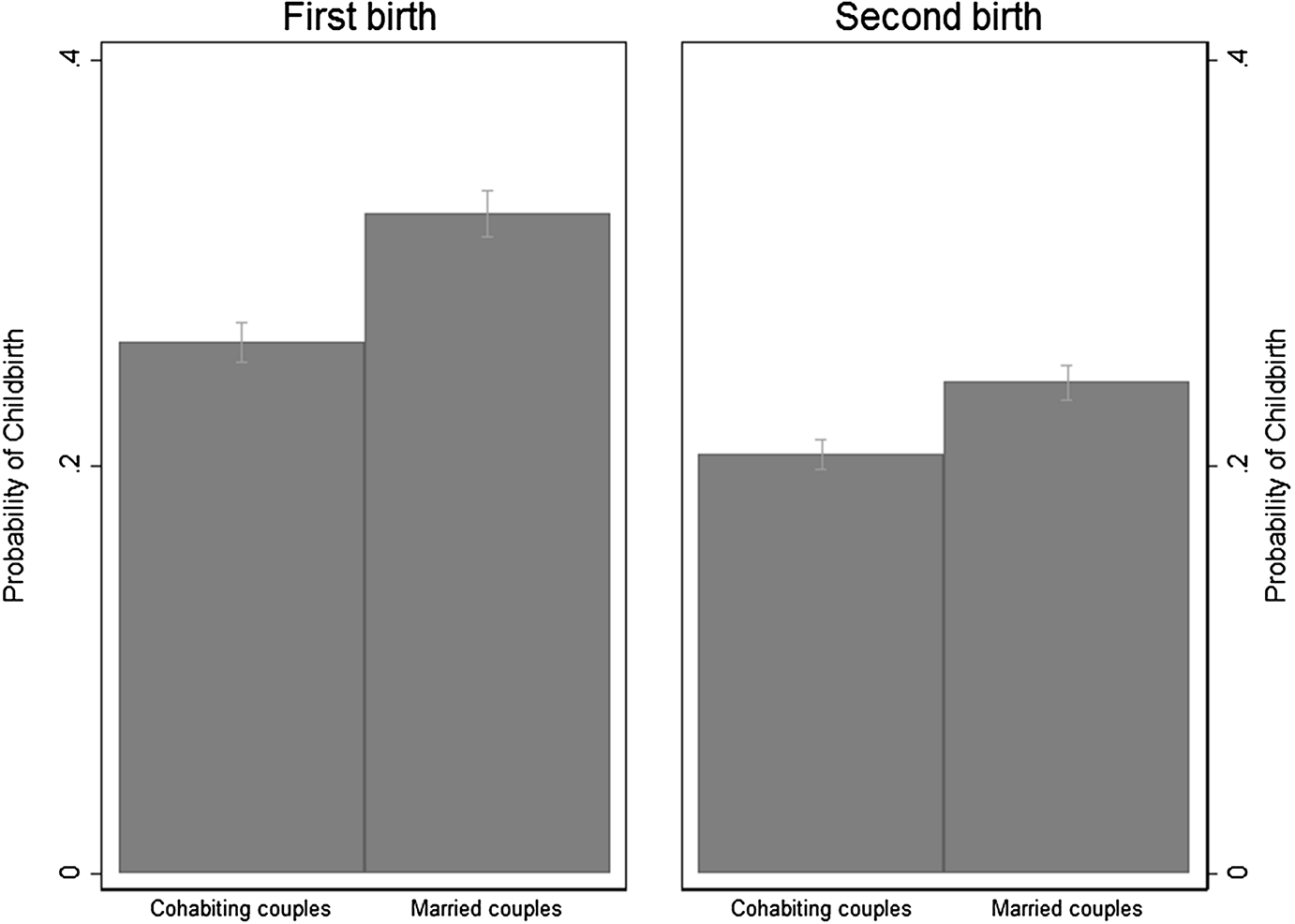
Norway-predicted probability of first and second birth by type of union (multi-process estimates)

To sum up, examining the predicted probabilities (Fig. 7) for both first- and second-birth transitions, Norwegian married women are more likely than cohabiting women to experience a birth. Further, Norway shows a higher probability of a second birth in both types of union.^15^ But remember that the youngest cohorts will not yet have completed their fertility trajectory. For both countries, we see that marriage is the preferred context for both first and second births. However, the mechanisms behind the same outcomes appear completely different.

## Discussion

Our starting point was whether cohabitation is increasingly a functional equivalent to formal marriage—at least as far as fertility behavior is concerned. This, we recall, is not what the postmodern ‘less family, more individualism’ version of the Second Demographic Transition thesis would expect (Lesthaeghe 2010). If the choice of cohabitation tends to reflect a weaker commitment to family life, it should also be associated with lower birth propensities. We opted for a Norway–Spain comparison since the two represent orthogonally different cultural and institutional contexts.

In Norway, cohabitation has been widely diffused, indeed institutionalized, for decades (Lappegaard and Norak 2015). Nowadays, among individuals aged 16–79, almost one in four couples are cohabiting (Statistics Norway). Spain, a clear exponent of lowest-low fertility, has experienced a rapid diffusion of cohabitation. Cohabitation rose from practically nil in 1990 to 17% of all unions in the mid-2000s.

In addition, overall fertility levels as well as partnership instability are greater in Norway. Around 50% of first births, indeed, occur within either premarital cohabitation or cohabitation. In Spain, from 1995 to 2010, non-marital births have increased from 11 to 35.5% (Dominguez-Folgueras and Castro-Martín 2013).

Childbearing is a measure of the degree to which cohabitation has gained strong social acceptance (Vitali et al. 2015). While Norway stands as a vanguard of family change, Spain is typically grouped within the traditionalist fold (Heuveline and Timberlake 2004; Esping-Andersen 2016). This, at first glance, would appear evident in terms of the evolution of cohabitation. In Norway, the latter has clearly attained normative status; in Spain it is very recent, and despite its rapid growth, we believed that it is unlikely that cohabitation would yet have attained broad acceptance as an alternative to marriage.

At first sight—and contradicting our expectations—we found that fertility patterns look quite similar across the two countries: the likelihood of first and second births is greater among married couples. However, our multi-process estimation revealed that behind this pattern of similarity lie distinct selection mechanisms. One advantage of multi-process multistate models is that they also provide an estimate of the underlying selection processes between different events; in this case, partnering and fertility. From the variance–covariance matrix, we observed that in Spain the correlation between cohabitation and fertility transitions is not significant, whereas it is in Norway. For Spain, this implies that those women with an above-average risk of childbirth do not show any significant correlation with those women that are more likely to enter into cohabitation. In contrast, in Norway women with an above-average risk of cohabitation also show an above-average risk of childbirth, meaning that cohabitation and fertility transitions are correlated.

Thanks to the multi-process multistate models, we also discovered that selection on unobservable time-invariant factors differs between country and by birth order. Further, the difference between single- and multi-process estimation showed that first births in Spain are greatly influenced by selection on time-invariant unobservables. For Norway, the same is the case for second births.

In the case of Spain, this is because we ‘cleansed’ the coefficient for risk of first birth, the positive correlation we find between fertility transitions and marriage; without allowing for this correlation, we would have overstated the effect of marriage on childbirth. Conversely, for Norway, the coefficient for the risk of a second birth is smaller in the multi-process than in the single-process estimation. This is partially in line with what we found in the variance covariance matrix. Accordingly, in the single-process approach the marriage effect is exaggerated because of selection on unobservables.

A possible explanation is that in Spain, a second birth represents an already selected group. Selection here is driven more by observable than unobservable characteristics. Thus, the difference between the single- and the multi-process estimates for the second-birth coefficient is negligible. In contrast, in Norway selection on unobservables is irrelevant because virtually everyone in any type of union will have a first child. When it comes to second births, which are less common, we observe that controlling for time-invariant unobservables plays a moderately important role.

On a more speculative note, can we expect this to continue? Some of the evidence suggests so, in particular considering the degree of normative acceptance that cohabitation has already attained in Spain. But we should also take into account the very different life course dynamics in the two societies. In contrast to Spain, Norwegian cohabitation is more dualistic, combining a large share of youth who most likely see it as a temporary arrangement, and more mature adults poised to start a family. Due to postponement, the Spanish enter into partnerships at a more mature age, pretty much across the board. And this, in turn, helps account for the surprising degree of stability within cohabiting partnerships. In a sense, Spanish cohabitation looks like a replica of marriage—but without the ceremony. In Norway, marriage has less to do with family formation and, as Perelli-Harris et al. (2014) argue, it appears more like a ceremony to celebrate a loving relationship.

Interpreting the relationship between type of union and fertility is not straightforward. As Dominguez-Folgueras and Castro-Martín (2013) show, Spanish cohabitation has diffused across all education levels within the more recent cohorts. However, even if cohabitation appears to enjoy broad social acceptance as a union option, this does not imply that it has gained normative acceptance for childbearing. Indeed, our results suggest that normative change as regards fertility behavior lags behind that of partnership choice. Norway, in contrast, exhibits a clearly different relationship between fertility and partnering. As emerges in Lappegard and Noak’s (2015) qualitative study, in Norway there is clearly no stigma attached to having children outside marriage. And yet, marriage continues to be viewed as the most natural context for fertility. These distinctly different normative contexts may, on a more speculative note, help account for the different country dynamics that lie behind apparently similar outcomes.

On a final note, multi-process estimation helps us deal with potential selection bias that is otherwise difficult to identify. Nevertheless, we should also remember that it is far from being a ‘cure-all’ remedy. We may have gotten a bit closer to identifying the logics that link partnering and childbearing choices, but we are clearly still far away from having fully opened the black box of all the possibly decisive mechanisms that drive both partnering and fertility. To this end, more in-depth qualitative research can potentially produce great value added.

## Electronic supplementary material

Below is the link to the electronic supplementary material.

Supplementary material 1 (PDF 237 kb)

## Footnotes

1 A partial exception is the USA where divorce rates are exceptionally high while cohabitation remains more infrequent (but is growing) (Cherlin 2009; Rindfuss and VandenHeuvel 1990; Smock 2000).

2 Perelli-Harris et al. (2014) find that the transition from cohabitation to marriage in Scandinavia is often a symbolic manifestation of a loving relationship.

3 Note, however, that until 2005 legal separation was a prerequisite for divorce in Spain. Our data treat both as equivalent to ‘divorce.’

4 Our portrait of Spanish cohabiters differs markedly from the evidence presented in Baizán et al. (2003). Analyzing 1995 data, i.e., in the very early stages of cohabitation, they found that it was strongly biased toward the higher educated and was associated with low fertility. As also highlighted in the Dominguez-Folgueras and Castro-Martín (2013) study (which is based on the same 2006 data we use), all this has changed in the past decades. The stable nature of Spanish cohabiters must be understood also in the context of late youth emancipation from the parental home, i.e., youthful 'shacking-up' is far less common than in Scandinavia.

5 Examining the age distribution, Norwegian cohabitation includes also a sizable group of youth who simply shack up. Since the under-25 s are far less likely to enter into parenthood (in both countries), this age bias must be kept in mind when we interpret fertility effects.

6 Note, however, that our data may overestimate first births within cohabitation. Spanish vital statistics, in fact, report that 30% of first births occur within cohabitation. This discrepancy may be due to the fact that we cannot observe completed fertility for the youngest cohort.

7 The time intervals are grouped into 6-month periods, and the risk of experiencing a transition is weighted by the number of months of the interval in which the event occurs (Steele et al. 2004). Monthly data were too demanding computationally speaking.

8 We take into account conception time.

9 We initially wanted to distinguish between married and previously cohabiting, cohabiting, cohabiting and previously married and married with a different partner. But this proved to be impossible due to inadequate N’s.

10 Most of the empirical evidence suggests that family-related preferences are quite volatile in late youth and very early adulthood, but become quite stable as individuals mature (see Alwin and Krosnick 1991; and also Axiin et al. 1994).

11 Women who remain childless are censored at their 45th birthday.

12 The detailed results from the multi-process fertility equations and also the diagnostic checks for the multi-process estimates are included in Appendix of ESM (Appendix Table A7, Table A8 and in Fig. A6 and Fig. A7).

13 In one of our robustness checks where we drop all people younger than 30, we find the same sign for this coefficient. However, the effect is slightly stronger (−0.40** in the single process and −0.47** in the multi-process).

14 In one of our robustness checks, where we drop all people younger than 30, we find the same sign for this coefficient. However, coefficients are slightly larger (0.62*** in the single process, 0.23** in the multi-process). Further, the difference between multi- and single process is greater for this sample, meaning that for first births there is more selection on unobservables. When it comes to second births, the coefficients are smaller than in the model shown (−0.11 in the single process and −0.29** in the multi-process). In the robustness check, the difference between the single and multi-process estimations is smaller. Additionally, when we exclude those under 30, the difference between Norway and Spain (for first births) diminishes considerably.

15 In order to test whether the difference by parity and type of union is statistically significant, we conducted a formal test of the significance of differences, as explained in Gelman and Stern (2006). For first births, the two countries show significant differences (i.e., confidence interval [−0.014, −0.039], But this is not the case for second births (i.e., C.I. [−0.001, 0.007]) despite the difference in the size of the estimates.
