# Supplementary material for: Partnership Choice and Childbearing in Norway and Spain
Source: Eur J Popul. 2017 Jun 22;34(3):367–86. doi: 10.1007/s10680-017-9432-6 (PMC6096890; doi:10.1007/s10680-017-9432-6)
Supplement: Supplementary file 1 — Supplementary material 1 (PDF 237 kb) [file 10680_2017_9432_MOESM1_ESM.pdf]

Appendix

(FIGURE A1 AND A2 ABOUT HERE)

The above graphs summarize the diagnostic checks for the multi-process estimation for, respectively, Spain and Norway. They help us to assess whether our model has reached convergence to the posterior distribution. Accordingly, we run the multi-process model with 300.000 iterations for Spain and 600.000 for Norway (the same diagnostics for the single model are available on request).

The first graph at the top on the left is the trace plot, and "it plots the generated values of the parameter against iteration number" (Advanced Multilevel Modelling using Markov chain Monte Carlo, p.44). In order to have a well-mixed chain it has to look like white noise. As we can see in both graphs the chains appear fairly well mixed. There are no evident fluctuations and the trend itself looks stable around a mean value. The second graph, at the top on the right side is the kernel density plot and it plots the posterior distribution. As we can see, for both countries, it is an almost symmetric distribution.

The two graphs on the second row are the autocorrelation (on the left side) and the partial autocorrelation (on the right side) functions. In general, they tell us how much the chains are correlated. In particular, in an independent chain we should observe that the two functions move to zero. In our case, the chain's auto correlation function is a slightly persistent but descendent, whereas the partial autocorrelation moves to zero. Finally, the last graph, on the bottom on the left side, represents a Monte Carlo standard error. This graph explains how much of the error term is due to the fact that we are using that simulation.

Fig.A1 Spain diagnostics checks

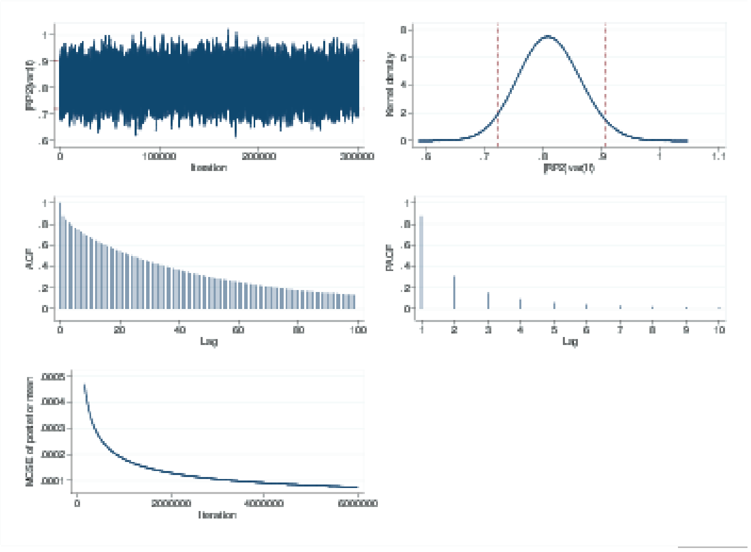

Fig.A2 Norway diagnostics checks

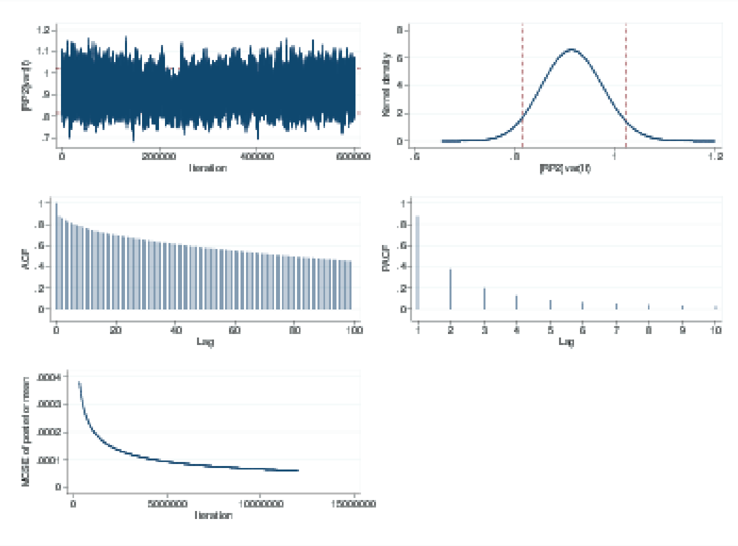

Table A 1 Descriptive statistics Spain from the fertility equation

	Person six-months					Number of events			
**Birth Cohort:**	Before 1959		After 1959			Before 1959		After 1959	
	Number	%	Number	%		Number	%	Number	%
First birth					Cohabiting	531	8.5	955	23.9
0	31188	52.9	19422	48.9	Married	5692	91.5	3040	76.1
1	27714	47.1	20298	51.1					
Second birth					Childless	183	2.9	683	17.1
0	33330	56.6	26578	66.9	First birth	3343	53.7	2114	52.9
1	25572	43.4	13142	33.1	Second birth	2697	43.3	1198	30.0
Total	**58902**	100.0	**39720**	100.0		**6223**	100.0	**3995**	100.0

Table A 2 Descriptive statistics Spain from the fertility equation

	Number of women				
**Birth Cohort**	Before 1959			After 1959	
Number of children	Number		%	Number	%
Childless	183		5.2	683	24.4
One child	646		18.3	916	32.7
Two children	2697		76.5	1198	42.8
Marital status					
Cohabiting	331		9.4	831	29.7
Married	3195		90.6	1966	70.3
Cohort					
1930-1939	1084	1960-1969	30.7	1434	51.3
1940-1949	1099	1970-1979	31.2	1113	39.8
1950-1959	1343	1980-1990	38.1	250	8.9
Highest Education Level of Respondent					
unknown edu	463		13.1	62	2.2
up to lower secondary	1598		45.3	224	8.0
upper secondary	1091		30.9	1546	55.3
Tertiary	374		10.6	965	34.5
Parental divorce before 18					
Yes	61		1.7	194	6.9
Total	**3526**		100.0	**2797**	100.0

Table A3 Descriptive statistics Norway from the fertility equation

	Person six-months					Number of events			
**Birth Cohort**	Before 1959		After 1959			Before 1959		After 1959	
	Number	%	Number	%		Number	%	Number	%
First birth					Cohabiting	555	10.8	2665	56.4
0	26777	58.4	22319	59.1	Married	4567	89.2	2058	43.6
1	19065	41.6	15431	40.9					
Second birth					Childless	273	5.3	911	19.3
0	27116	59.2	24585	65.1	First birth	2693	52.6	2231	47.2
1	18726	40.8	13165	34.9	Second birth	2156	42.1	1581	33.5
Total	**45842**	100.0	**37750**	100.0	Total	**5122**	100.0	**4723**	100.0

Table A4 Descriptive statistics Norway from the fertility equation

	Number of women				
**Birth Cohort**	**Before 1959**			After 1959	
Number of children	Number	%		Number	%
Childless	273	9.2		911	29.0
One child	538	18.1		652	20.8
Two children	2155	72.7		1579	50.3
Marital status					
Cohabiting	450	15.2		2116	67.3
Married	2516	84.8		1026	32.7
Cohort					
1930-1939	556	18.7	1960-1969	1442	45.9
1940-1949	1016	34.3	1970-1979	1179	37.5
1950-1959	1394	47.0	1980-1990	521	16.6
Highest Education Level of Respondent					
unknown edu	4	0.1		58	1.8
up to lower secondary	661	22.3		496	15.8
upper secondary	1410	47.5		1143	36.4
Tertiary	891	30.0		1445	46.0
Parental divorce before 18					
Yes	114	3.8		428	13.6
Total	**2966**	100.0		**3142**	100.0

Table A5 MCMC estimation for transition into partnership- Spain and Norway-multi process

	Spain		Norway	
**From single to marriage**	Coeff.	S.E.	Coeff.	
Constant	-7.14***	[0.17]	-9.11***	[0.34]
Duration	0.23***	[0.01]	0.30***	[0.03]
Duration squared	-0.00***	[0.00]	-0.01***	[0.00]
Birth Cohort (ref. 1960-69)				
1970-79	-0.30***	[0.07]	-0.43**	[0.14]
1980-90	0.29+	[0.16]	-0.67*	[0.28]
Level of education				
Missing	-0.61*	[0.24]	0.91**	[0.34]
Tertiary	-1.30***	[0.16]	-1.03***	[0.21]
Upper secondary	-0.45***	[0.12]	-0.72***	[0.20]
Parental Divorce	-0.17	[0.16]	-0.83***	[0.25]
Previous partnership	-0.49+	[0.27]	0.03	[0.25]
Age of current children				
Between 0 and 5	0.60***	[0.15]	0.02	[0.25]
Between 5 and 18	-0.50*	[0.24]	-0.54+	[0.30]
Older than 18	-160.55	[127.88]	-1.53*	[0.72]
**From single to cohabitation**				
Constant	-8.84***	[0.26]	-6.06***	[0.09]
Duration	0.14***	[0.01]	0.19***	[0.01]
Duration squared	0.00	[0.00]	-0.00***	[0.00]

Birth Cohort (ref. 1960-69)				
1970-79	1.17***	[0.11]	0.32***	[0.05]
1980-90	3.08***	[0.18]	0.96***	[0.07]
Level of Education				
Missing	0.13	[0.32]	-1.58***	[0.20]
Tertiary	-0.50**	[0.19]	-0.67***	[0.07]
Upper secondary	-0.27	[0.18]	-0.23***	[0.06]
Parental Divorce	1.05***	[0.15]	0.21***	[0.06]
Previous partnership	3.36***	[0.20]	1.26***	[0.06]
Age of current children				
Between 0 and 5	0.21	[0.16]	-0.19*	[0.07]
Between 5 and 18	-0.18	[0.18]	-0.64***	[0.08]
Older than 18	-0.26	[0.38]	-0.81***	[0.20]
Observations	238208		274898	

Table A6 MCMC estimation for cohabitation to marriage- cohabitation to separation transition -Spain and Norway- multiprocess

	**Spain**		**Norway**	
**From cohabitation to marriage**	Coeff.	S.E.	Coeff.	S.E.
Constant	-4.71***	[0.41]	-5.24***	[0.19]
Duration	-0.01	[0.03]	0.12***	[0.01]
Duration squared	-0.00	[0.00]	-0.00***	[0.00]
Birth Cohort (ref. 1960-69)				
1970-79	-0.26+	[0.15]	-0.45***	[0.07]
1980-90	-0.87**	[0.28]	-2.24***	[0.24]
Level of Education				
Missing	-1.27*	[0.59]	-0.57	[0.37]
Tertiary	0.09	[0.26]	-0.06	[0.09]
Upper secondary	-0.07	[0.25]	-0.13	[0.09]
Parental Divorce	-0.16	[0.21]	-0.19*	[0.09]
Migrant	0.09	[0.17]	0.22	[0.14]
Previous partnership	-1.01***	[0.25]	-0.28***	[0.08]
Previous children	-0.60***	[0.13]	-0.20***	[0.05]
**From cohabitation to separation**				
Constant	-7.30***	[0.63]	-5.31***	[0.20]
Duration	0.17***	[0.04]	0.09***	[0.02]
Duration squared	-0.00**	[0.00]	-0.00***	[0.00]
Birth Cohort (ref. 1960-69)				

1970-79	0.61*	[0.24]	0.17^{*}	[0.07]
1980-90	0.43	[0.40]	0.27^{**}	[0.10]
Level of Education				
Missing	-0.15	-0.15	0.06	[0.34]
Tertiary	0.01	0.01	-0.02	[0.09]
Upper secondary	-0.02	-0.02	-0.04	[0.09]
Parental Divorce	0.00	0.00	0.48^{***}	[0.08]
Migrant	-0.11	-0.11	-0.08	[0.16]
Previous partnership	-0.47	-0.47	-0.45^{***}	[0.09]
Previous children	-0.59***	-0.59***	-0.65^{***}	[0.06]
Observations	238208	238208	274898	

Table A7 MCMC estimation for childbirth within partnership (Single-process versus Multi-process estimates) - Spain

	Single Process		Multi Process	
**Childless women**				
Constant	-4.20***	[0.14]	-3.72***	[0.08]
Partnership duration				
(ref. First two years)				
Up to 3 years	0.67***	[0.06]	0.68***	[0.04]
Up to 6 years	0.71***	[0.08]	0.53***	[0.06]
Morethan6years	0.86***	[0.11]	0.29***	[0.07]
Highest education				
(ref. up to lower secondary)				
Unknown	0.11	[0.22]	-0.23**	[0.08]
Upper secondary	-0.14	[0.11]	-0.20***	[0.05]
Tertiary	-0.60***	[0.12]	-0.63***	[0.06]
Birth cohort				
(ref. 1960-1969)				
1970-79	-0.26***	[0.06]	-0.51***	[0.06]
1980-90	-0.39**	[0.15]	-0.64***	[0.14]
Background characteristics				
Parental Divorce	-0.10	[0.13]	-0.15	[0.11]
Not Migrant	0.27**	[0.09]	0.15+	[0.08]
Partnership dissolution	0.15	[0.18]	-0.59***	[0.18]
Type of union				

Marriage (ref. cohab.)	1.07***	[0.08]	0.62***	[0.07]
Age at partnership				
(ref. 15-20)				
21-25	-0.40***	[0.08]	-0.07	[0.05]
26+	-0.52***	[0.08]	-0.25***	[0.06]
**One child women**				
Constant	-6.12***	[0.20]	-5.66***	[0.11]
Partnership duration				
(ref. First two years)				
Up to 3 years	-0.30*	[0.15]	0.35***	[0.08]
Up to 6 years	0.29*	[0.14]	1.09***	[0.09]
More than 6 years	0.80***	[0.14]	1.49***	[0.09]
Highest education				
(ref. up to lower secondary)				
Missing	0.28	[0.29]	0.24**	[0.09]
Upper secondary	-0.07	[0.14]	-0.16**	[0.06]
Tertiary	0.29+	[0.16]	0.14+	[0.07]
Birth cohort				
(ref. 1960-69)				
1970-79	0.31***	[0.09]	0.16+	[0.08]
1980-90	0.50+	[0.27]	0.19	[0.26]
Background characteristics				
Parental Divorce	0.13	[0.19]	0.17	[0.14]
Not Migrant	-0.07	[0.13]	-0.04	[0.10]

Partnership dissolution	1.36***	[0.25]	1.55***	[0.22]
Type of union				
Marriage (ref. cohab)	-0.51***	[0.13]	-0.50***	[0.08]
Age at partnership (ref. 15-20)				
21-25	0.21+	[0.11]	-0.03	[0.06]
26+	0.42***	[0.11]	0.11+	[0.06]
Observations	39720		238208	

Standard errors in brackets + p<0.1, * p<0.05, ** p<0.01, *** p<0.001

*six month intervals

Table A8 MCMC estimation for childbirth within partnership (Single-process versus Multi-process estimates) - Norway

	Single Process		Multi Process	
**Childless women**				
Constant	-3.94***	[0.09]	-3.57***	[0.07]
Partnership duration				
(ref. First two years)				
Up to 3 years	0.56***	[0.06]	0.56***	[0.04]
Up to 6 years	0.55***	[0.08]	0.54***	[0.06]
Morethan6years	0.75***	[0.10]	0.44***	[0.08]
Highest education				
(ref. up to lower secondary)				
Unknown	-0.17	[0.23]	-0.10	[0.24]
Upper secondary	-0.18*	[0.09]	-0.17**	[0.06]
Tertiary	-0.32***	[0.09]	-0.33***	[0.06]
Birth cohort				
(ref. 1960-1969)				
1970-79	-0.27***	[0.06]	-0.35***	[0.06]
1980-90	-1.43***	[0.13]	-1.82***	[0.13]
Background characteristics				
Parental Divorce	-0.05	[0.08]	-0.06	[0.08]
Not Migrant	-0.22+	[0.11]	-0.25**	[0.09]
Partnership dissolution	0.17	[0.07]	-0.19*	[0.08]
Type of union				

Marriage (ref. cohab.)	0.70***	[0.06]	0.66***	[0.05]
Age at partnership				
(ref. 15-20)				
21-25	-0.07	[0.07]	-0.30***	[0.05]
26+	-0.14*	[0.07]	-0.49***	[0.06]
**One child women**				
Constant	-5.05***	[0.12]	-5.09***	[0.09]
Partnership duration				
(ref. First two years)				
Up to 3 years	-0.48***	[0.10]	0.00	[0.07]
Up to 6 years	0.15	[0.10]	0.74***	[0.07]
More than 6 years	0.37***	[0.11]	1.07***	[0.08]
Highest education				
(ref. up to lower secondary)				
Missing	-0.19	[0.35]	-0.36	[0.33]
Upper secondary	0.15	[0.11]	0.05	[0.07]
Tertiary	0.14	[0.11]	0.11	[0.07]
Birth cohort				
(ref. 1960-69)				
1970-79	0.30***	[0.08]	0.39***	[0.07]
1980-90	1.10***	[0.27]	1.00***	[0.27]
Background characteristics				
Parental Divorce	0.10	[0.11]	0.07	[0.10]
Not Migrant	-0.23	[0.15]	-0.11	[0.11]

Partnership dissolution	0.16*	[0.08]	0.33***	[0.08]
Type of union				
Marriage (ref. cohab)	-0.17*	[0.08]	-0.53***	[0.07]
Age at partnership (ref. 15-20)				
21-25	0.19*	[0.10]	0.18**	[0.06]
26+	0.42***	[0.10]	0.49***	[0.07]
Observations	37750		274898	

Standard errors in brackets + p<0.1, * p<0.05, ** p<0.01, *** p<0.00
